# snRNA‐Seq Unveils Cell‐Type‐Specific Immune Dynamics in *Arabidopsis* During Pinewood Nematode Infection

**DOI:** 10.1111/mpp.70136

**Published:** 2025-08-18

**Authors:** Meiling Wang, Xiehai Song, Zhiyuan Jiao, Jiashu Zhang, Yue Sang, Wei Li

**Affiliations:** ^1^ State Key Laboratory of Tree Genetics and Breeding, National Engineering Research Center of Tree Breeding and Ecological Restoration, College of Biological Sciences and Technology Beijing Forestry University Beijing China; ^2^ Shandong Key Laboratory of Precision Molecular Crop Design and Breeding, Peking University Institute of Advanced Agricultural Sciences Shandong Laboratory of Advanced Agricultural Sciences in Weifang Weifang Shandong China

**Keywords:** immune dynamics, pinewood nematode, regional infection response, single‐nucleus transcriptomics

## Abstract

The alterations in gene expression levels in response to the pathogens are pivotal in determining pathogenicity or susceptibility. However, the cell‐type‐specific interaction mechanism between the pinewood nematode (PWN) and its hosts remains largely unexplored and poorly understood. Here, we employed single‐nucleus RNA sequencing (snRNA‐seq) with PWN‐infected *Arabidopsis* leaves to dissect the heterogeneous immune responses. We identified four major cell types, each exhibiting distinct immune responses during infection by PWNs. Subcluster analyses uncovered dynamic shifts in immune‐active subpopulations within mesophyll and epidermal cells. Notably, *AtWRKY70* positively regulated plant defence against PWNs by suppressing the promoter activity of *AtPNP‐A* in a salicylic acid‐dependent manner. This study not only provides novel mechanistic insights into plant gene regulation during PWN infection, but also offers feasible references for future investigations of host–PWN interactions, with particular relevance to the identification of pine tree resistance genes against this pathogen.

## Graphical abstract

Single‐nucleus RNA sequencing (snRNA‐seq) revealed significant cell‐type‐specific differences in pinewood nematode (PWN)‐induced transcriptional reprogramming and identified the critical role of the AtWRKY70‐AtPNP‐A transcriptional regulatory module during PWN infection.

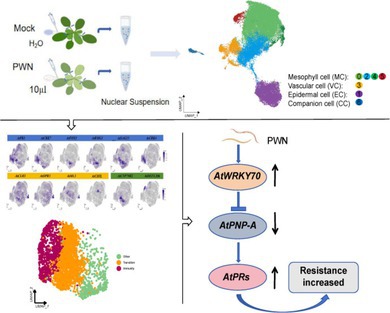

## 1 Introduction

The pinewood nematode (PWN, *Bursaphelenchus xylophilus*) is a globally significant quarantine pest that inflicts irreversible damage to the vascular systems of its host trees, leading to host mortality within months (Ye and Wu 2022; Back et al. 2024; Tong et al. 2024). The resulting devastation has caused substantial economic losses and poses a severe threat to pine forest resources and ecological security (Kim et al. 2020; Liu, Su, et al. 2023; Liu, Yang, et al. 2023). Investigating the defence mechanisms of host pine trees against PWN infection is crucial for understanding the intrinsic causes of pine tree mortality and identifying effective control strategies (Wang et al. 2023; Modesto et al. 2022; Liu, Zhang, Fang, et al. 2022; Liu, Zhang, Xin, et al. 2022). However, research on pine trees as host plants has been limited, and the molecular‐level understanding of the interactions between pine trees and pinewood nematodes remains incomplete.

There is natural susceptibility to pine wilt disease in at least 51 *Pinus* species and 16 non‐*Pinus* arboreal taxa worldwide (Ye and Wu 2022). Recent experimental inoculations demonstrated that PWN can colonise *Arabidopsis thaliana* , inducing precocious symptomatology analogous to coniferous host responses (Zhao et al. 2013; Xie et al. 2022). This breakthrough establishes *Arabidopsis* as a tractable model system for interrogating PWN–host molecular interactions. *A. thaliana* has been extensively used as a model system for deciphering molecular mechanisms underlying plant–parasitic nematode interactions (Hamamouch et al. 2020; Noureddine et al. 2022; Radakovic et al. 2018; Warmerdam et al. 2020). Therefore, the molecular mechanism of *Arabidopsis* immune response to PWNs could be further explored.

Previous studies on the molecular response mechanisms of hosts to PWNs have primarily focused on identifying putative resistance genes through transcriptomic and genomic analyses (Gaspar et al. 2020; Hirao et al. 2012; Liu et al. 2017; Nose and Shiraishi 2011; Shin et al. 2009). However, the functional characterisation of these genes and the complex interaction mechanisms between PWNs and their host trees require further exploration (Xu et al. 2016; Hu and Wu 2018). Plant–pathogen interactions are highly dynamic processes that result in heterogeneous cellular responses (Zhu et al. 2023). Cell‐type‐specific characterisation is essential for a deeper understanding of plant responses to environmental stimuli (Bezrutczyk et al. 2021; Kim et al. 2021; Xia et al. 2022). Nevertheless, the cell‐type‐specific immune responses of host plants to PWNs remain poorly characterised. Single‐cell RNA sequencing (scRNA‐seq) enables the interrogation of populations at the single‐cell level and genome‐wide scale, allowing for the analysis of transcriptomes from diverse cell types and states (Libault et al. 2017; Seyfferth et al. 2021). The application of scRNA‐seq in plants has provided new insights into cell function and development across different tissues (Denyer et al. 2019; Procko et al. 2022; Shahan et al. 2022; Zhang et al. 2021). Additionally, scRNA‐seq has been used to study plant responses to biotic and abiotic stresses (Jean‐Baptiste et al. 2019; Bai et al. 2022; Zha et al. 2023; Liang et al. 2023; Liu, Su, et al. 2023; Liu, Yang, et al. 2023; Sun et al. 2022; Zhu et al. 2023). scRNA‐seq enables the elucidation of heterogeneous host responses to infection at single‐cell resolution, providing unprecedented insights into plant immunity.

Here, we constructed the single‐cell transcriptional landscape of *Arabidopsis* leaves during PWN infection. Based on the expression patterns of cell lineages in response to PWN infection, we analysed pathways and genes associated with PWN resistance in *Arabidopsis*. We found that salicylic acid (SA)‐mediated immune responses are pivotal for conferring resistance against PWNs in *Arabidopsis*. Moreover, we demonstrated that AtWRKY70 suppresses the promoter activity of *AtPNP‐A* to inhibit PWN infection through an SA‐dependent manner. This study provides unprecedented insights into the molecular mechanisms underlying plant resistance to PWNs at single‐cell resolution and offers a foundation for future strategies in the control and quarantine of pine wilt disease.

## 2 Results

### 2.1 Single‐Nucleus RNA Sequencing Profiling of *Arabidopsis* Leaves Infected With PWNs

**FIGURE 1:**
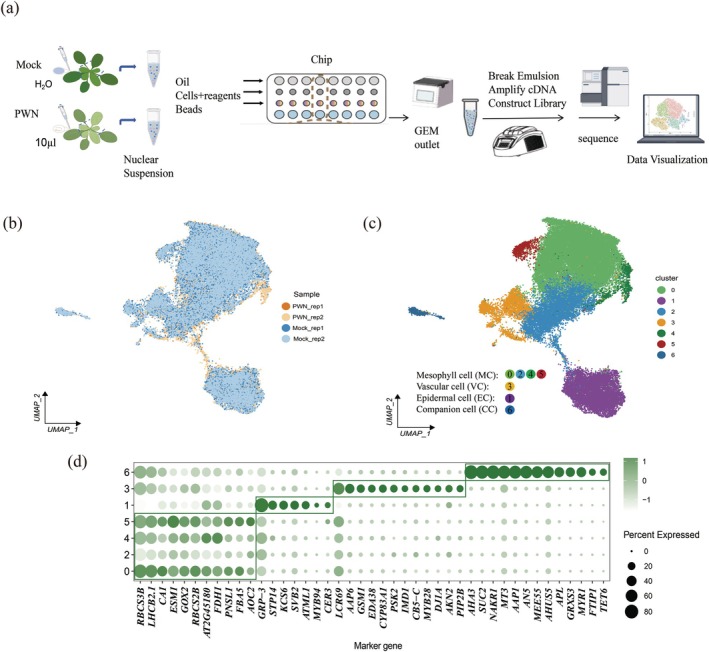
Single‐cell RNA‐seq profiling of *Arabidopsis* infected with pinewood nematodes (PWNs). (a) Overview of the scRNA‐seq experiment. (b) Integration of four single‐nucleus datasets. (c) UMAP visualisation of seven identified cell clusters. (d) Dot plot of cell type‐specific marker genes. The size of the dot represents the percentage of cells expressing the gene in each cluster. The colour intensity represents the level of gene expression.

It has been reported that *Arabidopsis* can act as a host plant for studying plant–PWN interactions (Zhao et al. 2013). To delineate cell type‐specific transcriptional dynamics during PWN infection, 22‐day‐old *A. thaliana* plants were inoculated with PWNs. Symptomatic leaf samples were collected 7 days post‐inoculation (dpi) for single‐nucleus RNA sequencing (snRNA‐seq). Leaves from plants at an identical developmental stage, mock‐inoculated with distilled water, served as controls. Both PWN‐inoculated (PWN_rep1, PWN_rep2) and mock‐inoculated (Mock_rep1, Mock_rep2) plants included two biological replicates. Following nuclear isolation, snRNA‐seq libraries were generated using the BMK DG1000 platform (Figure 1a). After removing doublets and low‐quality nuclei, we obtained transcriptomes for 43,531 cells across all four samples, detecting the expression of 17,522 genes with a median of 792 genes per cell (Table S1). Through the analysis of the uniform manifold approximation and projection (UMAP) plot of each sample, it was found that there was a high degree of consistency among the processing repetitions (Figure 1b; Figure S1). After integrating the four datasets using scVI, we identified seven distinct cell clusters. For cluster annotation, we used previously reported marker genes for *Arabidopsis* (Kim et al. 2021; Lopez‐Anido et al. 2021; Procko et al. 2022; Xia et al. 2022; Liu, Zhang, Fang, et al. 2022; Liu, Zhang, Xin, et al. 2022; Han et al. 2023; Zhu et al. 2023), successfully identifying four major cell types (Figure 1c, Table S2), including mesophyll cells (MC) (clusters 0, 2, 4, 5), vascular cells (VC) (cluster 3), epidermal cells (EC) (cluster 1) and companion cells (CC) (cluster 6). In order to verify the accuracy of our annotated cell types, we compared the expression levels of marker genes in each cell type and also conducted analysis of differentially expressed genes (DEGs) among the four cell types. The results showed that each major cell type expressed a unique set of marker genes corresponding to the cell type (Figure 1d). In summary, our results indicate the reliability of the experimental and computational procedures for single‐cell transcriptomic analyses.

### 2.2 PWN‐Induced Transcriptomic Changes Vary Among Cell Types

**FIGURE 2:**
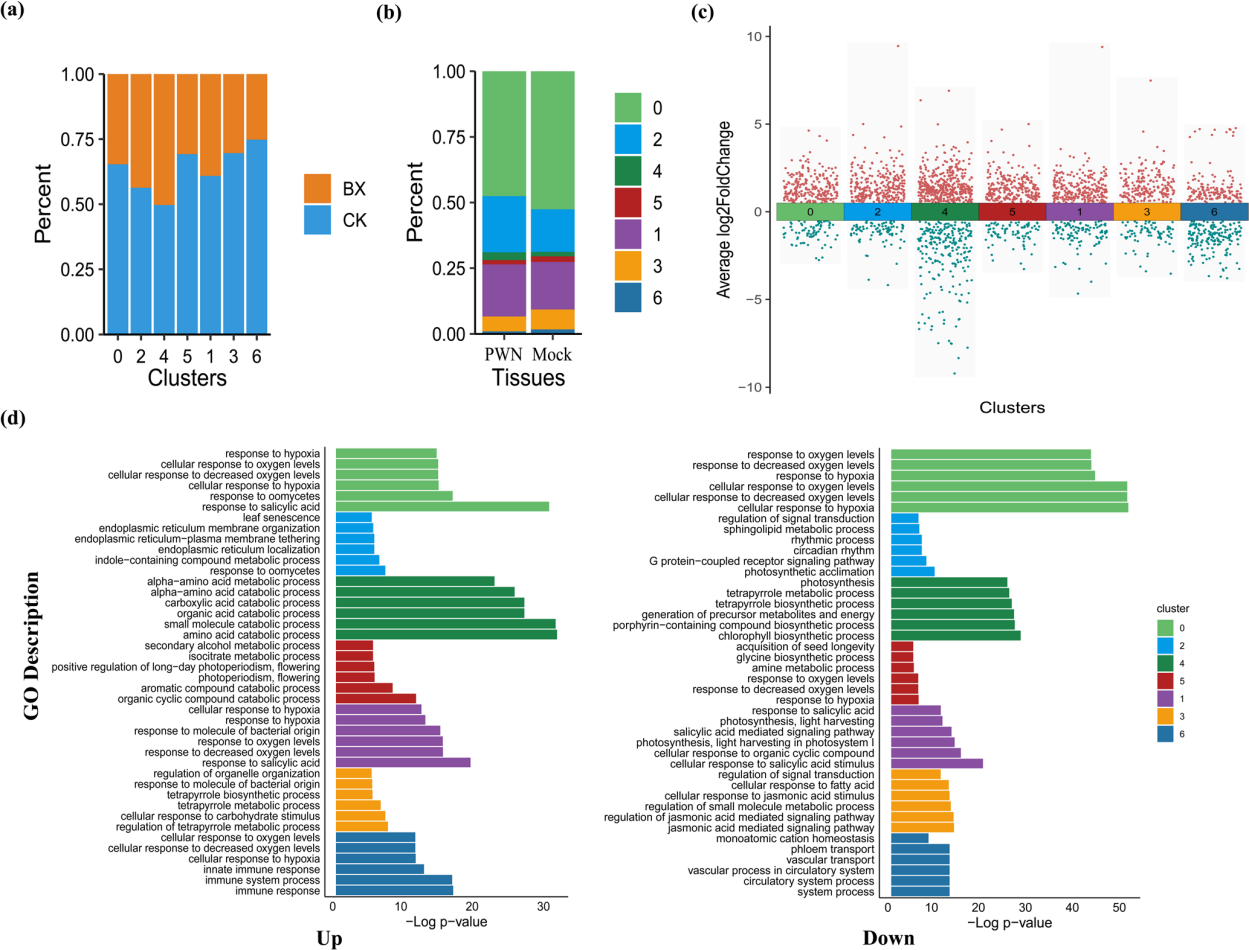
Single‐cell transcriptome analysis of Mock‐inoculated and pinewood nematode (PWN)‐inoculated *Arabidopsis* leaves. (a) The relative proportion cell types of Mock (CK) and PWN (BX) treatments in clusters. (b) The proportion of each cluster in the Mock and PWN treatments. (c) Number and foldchange (FC) differences of differentially expressed genes (DEGs) in each cluster with PWN treatment. Dots indicate the number of DEGs (|log_2_FC ≥ |0.5|, *p* ≤ 0.01) in PWN versus Mock in each cluster, log_2_FC indicates fold of difference, positive values indicate high expression in PWN and negative values indicate high expression in Mock treatment. (d) The GO terms of *Arabidopsis* leaves in response to PWNs.

To investigate changes in transcript levels in cells of the PWN‐infected sample, the single‐cell transcriptomic data were analysed by comparing the responses of various cell types to PWN infection. When PWNs infect *Arabidopsis* leaves, they do not induce changes in cell types but alter the proportions of different cell types (Figure 2a). Specifically, epidermal cells (cluster 1) and vascular cells (clusters 3 and 6) exhibit opposite trends (Figure 2b). During PWN infection, the proportion of epidermal cells significantly increases, while the proportion of vascular cells decreases. To investigate the level of heterogeneity after PWN infection, GO annotation analyses were conducted by comparing DEGs across different cell types (Figure 2c, Table S3). Intriguingly, the immune‐related genes were predominantly found in a single cell type. In addition, we observed distinct gene expression patterns across different cell types during PWN infection. For instance, the up‐regulated genes in companion cells were primarily associated with immune responses, whereas the down‐regulated genes in vascular cells were mainly related to the jasmonic acid (JA) signalling pathway. In epidermal cells, DEGs were significantly enriched in the salicylic acid (SA) signalling pathway (Figure 2d,e). Mesophyll cells displayed coordinated induction of reactive oxygen species (ROS)‐associated genes and secondary metabolism enzymes. These findings indicate the specialised roles of different plant cell types in response to PWN infection. Spatial regulation of immune pathways enables integrated host responses to biotic challenges.

### 2.3 Fine Dissection of PWN‐Induced Immune Cell States

**FIGURE 3:**
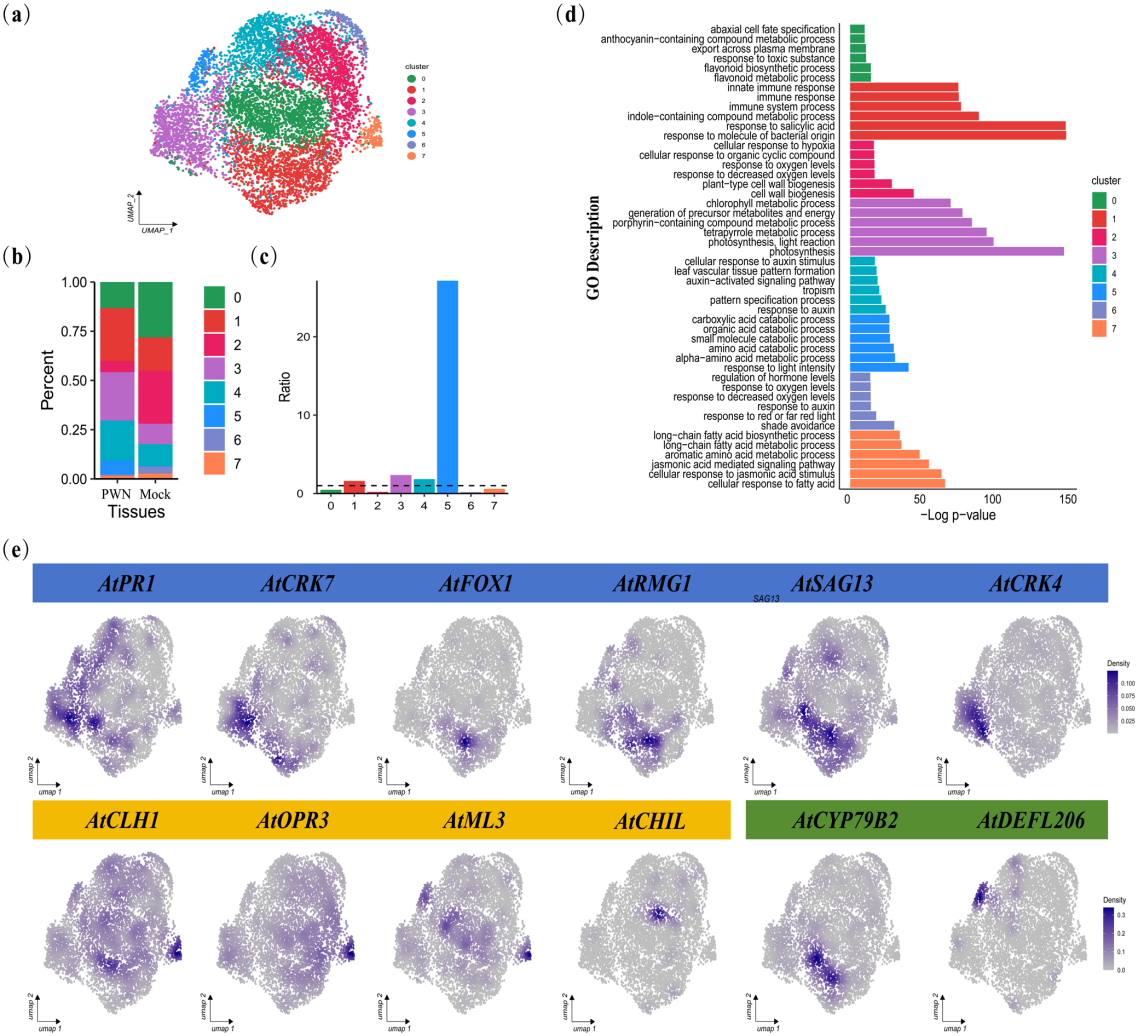
Specific responses of epidermal cell subtypes to piewood nematodes (PWNs). (a) UMAP visualisation of epidermal cell subtypes. (b) The proportion of each cluster in Mock‐ and PWN‐treated samples. (c) Ratio of each cell subtype in PWN and Mock samples. (d) GO enrichment analysis of genes within each cluster. (e) UMAP visualisation of expression patterns of immune‐related and susceptibility genes. Immune marker genes are represented by blue blocks, susceptibility marker genes by yellow blocks and insect resistance marker genes by green blocks.

To resolve cell type‐specific immune responses, we performed secondary clustering on major cell populations. EC partitioned into eight subtypes (Figure 3a), MC yielded seven (Figure S4a), while CC and VC resolved into four and six subtypes, respectively (Figure S2). Although plant immunity genes were expressed across all cell types, the primary immune‐active genes were predominantly concentrated in the mesophyll and epidermal cell types (Figure 2c), consistent with prior reports (Nobori et al. 2025). Further analyses of the epidermal subtypes showed that the 1, 3, 4 and 5 clusters significantly increased in PWN samples, whereas the 0, 2, 6 and 7 clusters decreased (Figure 3b,c). GO enrichment analyses revealed that cluster 1 was specifically enriched in immune response pathways, whereas cluster 7 was primarily enriched in the JA signalling pathway (Figure 3d), indicating distinct functional roles during PWN infection. In addition, UMAP expression profiling of marker genes revealed that disease resistance‐related genes, such as pathogenesis‐related protein gene 1 (*PR1*), senescence‐associated gene 13 (*SAG13*), cysteine‐rich RLK (RECEPTOR‐like protein kinase) 4 (*CRK4*), cysteine‐rich RLK7 (*CRK7*), fad‐linked oxidoreductase 1 (*FOX1*), and resistance methylated gene 1 (*RMG1*), were highly expressed in clusters 1, 3 and 5. In contrast, susceptibility marker genes, such as chlorophyllase 1 (*CLH1*), oxophytodienoate‐reductase 3 (*OPR3*) and md2‐related lipid recognition 3 (*ML3*), were specifically expressed in clusters 0 and 7. Cytochrome P450 79B2 (*CYP79B2*) and defensin‐like protein 206 (*DEFL206*), which confer insect resistance in *Arabidopsis* (Clay et al. 2009; Hawamda et al. 2022), in clusters 1 and 5 further substantiated the functional importance of clusters 1, 3 and 5 during PWN infection (Figure 3e). To verify the identity of these genes, we assessed their expression using transcriptomic data and reverse transcription‐quantitative real‐time PCR (RT‐qPCR). This analysis revealed that defence‐related genes were up‐regulated following PWN infection, particularly after 7 days, while susceptibility‐associated genes were down‐regulated (Figure S3); collectively supporting the reliability of our immune‐responsive cell classification. The cell type‐specific expression patterns of these genes elucidate potential mechanisms for systemic propagation of PWN resistance signals.

**FIGURE 4:**
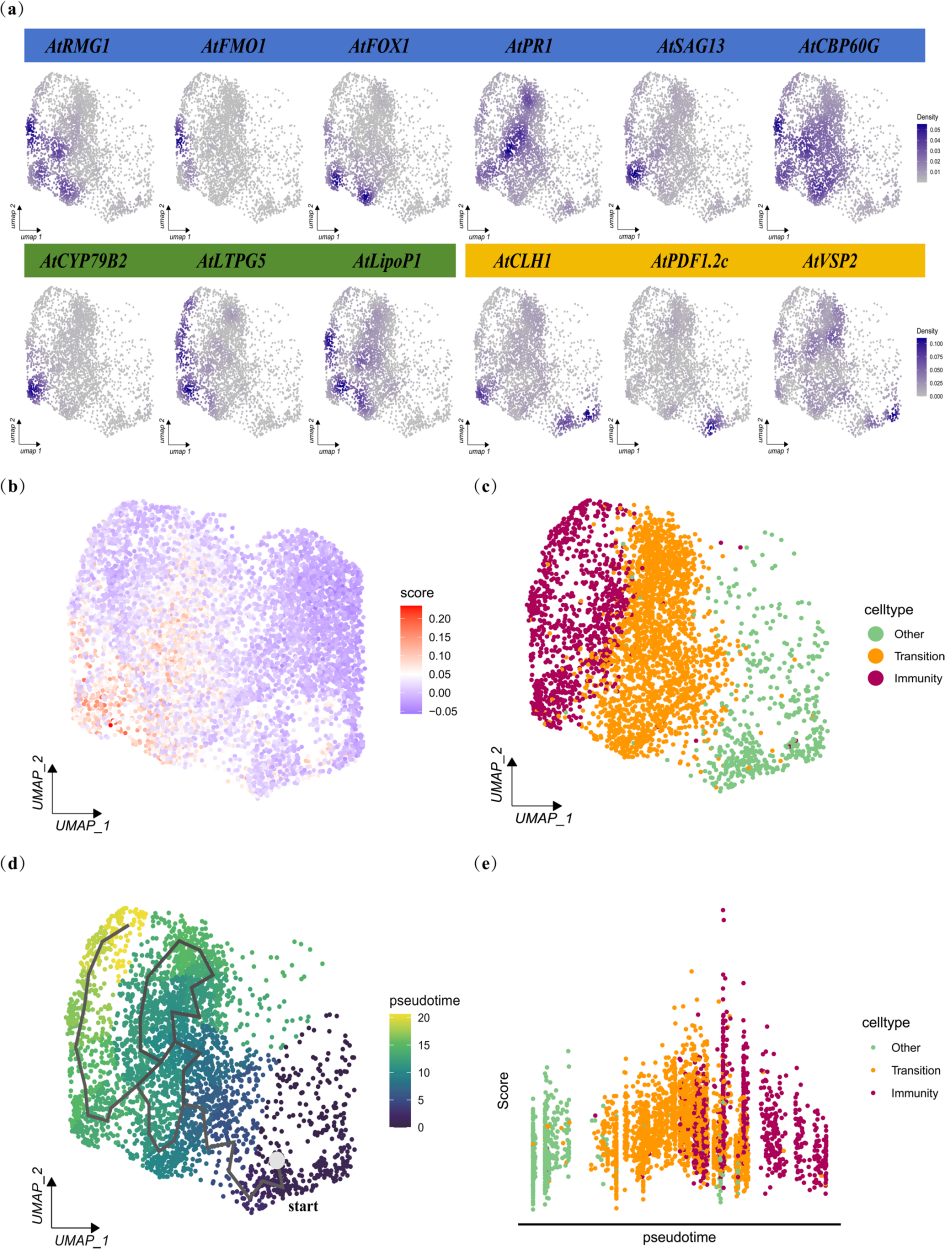
Single‐nucleus RNA sequencing (snRNA‐seq) reveals the continuum of immune responses induced by pinewood nematodes (PWNs) in mesophyll cells. (a) UMAP visualisation of expression patterns of immune‐related and susceptibility genes in mesophyll cells. (b) UMAP illustrating the extent of responses to PWN infection. The immune response score, calculated as a gene expression module (STAR Methods) based on cell‐specific expression of known immune‐related genes, is shown. Blue (negative values) indicates a more susceptible‐like state, while red (positive values) indicates a more immune‐like state. (c) UMAP after cell classification. Cells are coloured according to their cluster membership: green cells represent non‐responsive mesophyll cells (others), red cells belong to the immune cluster and orange cells correspond to the transitional cluster. (d) Pseudotime trajectory of mesophyll cells demonstrating a directional transition from low to high immune activity. (e) PWN‐induced signatures across pseudotime.

The subtypes of mesophyll cells analyses revealed that the cell numbers of clusters 0, 3 and 5 increased while clusters 1, 2, 4 and 6 decreased in the PWN samples (Figure S4b,c). GO enrichment analyses showed that clusters 0 and 5 were mainly associated with immune responses and systemic acquired resistance (SAR), while clusters 4 and 6 were primarily enriched in the JA signalling pathway (Figure S4e). To verify the identity of the cluster, we identified genes specifically expressed in a single cluster (Figure S4d), demonstrating the reliability of the classification. Next, we analysed the expression levels of plant defence genes, *RMG1*, flavin‐dependent monooxygenase 1 (*FMO1*), *FOX1*, *SAG13* calmodulin binding protein 60‐like.g (*CBP60g*) and *PR1*—along with insect resistance genes *CYP79B2*, glycosyl phosphatidylinositol‐anchored lipid protein transfer 5 (*LTPG5*) and lipoprotein 1(*LipoP1*) in clusters 0, 2, 3 and 5. Conversely, the susceptibility gene *CLH1* exhibited increased expression in clusters 4 and 6 (Figure 4a; Figure S5). Among the DEGs in response to PWN infection, we identified three down‐regulated genes: chalcone isomerase like (*CHIL*), plant defensin 1.2c (*PDF1.2c*) and vegetative storage protein 2 (*VSP2*) (Figure S3; Figure S5). UMAP projection revealed their specific expression in non‐immune cell populations (Figures 3e and 4a), suggesting a negative regulatory role during PWN infection. Paradoxically, established functions indicate that *PDF1.2c* exhibits antifungal activity (Hao et al. 2023), *VSP2* is implicated in anti‐insect defence (Zhao et al. 2023) and *CHIL* catalyses secondary metabolism enhancing plant resistance (Hiruma et al. 2011). Our findings demonstrate their association with PWN susceptibility, suggesting functional divergence and their potential as candidate susceptibility marker genes for the host plant response to PWN.

Then, we calculated the immune response score based on the expression modules of the immune genes and found that the expression of genes induced by PWN infection in clusters 2 and 5 was generally higher than in other clusters (Figure 4b, Table S5). Thus, these clusters were identified as immune cell types. Although some defence‐related genes were up‐regulated in clusters 0 and 3, their expression levels were lower than those in clusters 2 and 5. We defined them as transition cell types, and clusters 1, 4 and 6 were defined as non‐immune cells (Figure 4c). To predict the developmental trajectory of PWN‐responsive cell types, we performed pseudotime analyses using Monocle 3. The trajectory primarily started with cluster 4, followed by a transition through clusters 0 and 3 and finally reached clusters 2 and 5 (Figure 4d). When overlaid on pseudotime, gene scores were significantly induced in both transition and immune cell types (Figure 4e), reflecting the transcriptional reprogramming of mesophyll cells inoculated with PWNs, uncovering heterogeneous immune states within the population. These results indicate that immune activities in plant cells exhibit a compartmentalised nature, and different cell types function through distinct immune pathways. Putative cell population‐specific immunity genes were identified based on expression differentials between defined cell types and all other cells. This finding underscores the necessity of single‐cell resolution for mechanistic dissection of immune responses.

### 2.4 Identification of Core Transcription Factors in *Arabidopsis* Leaf Responses to PWNs

**FIGURE 5:**
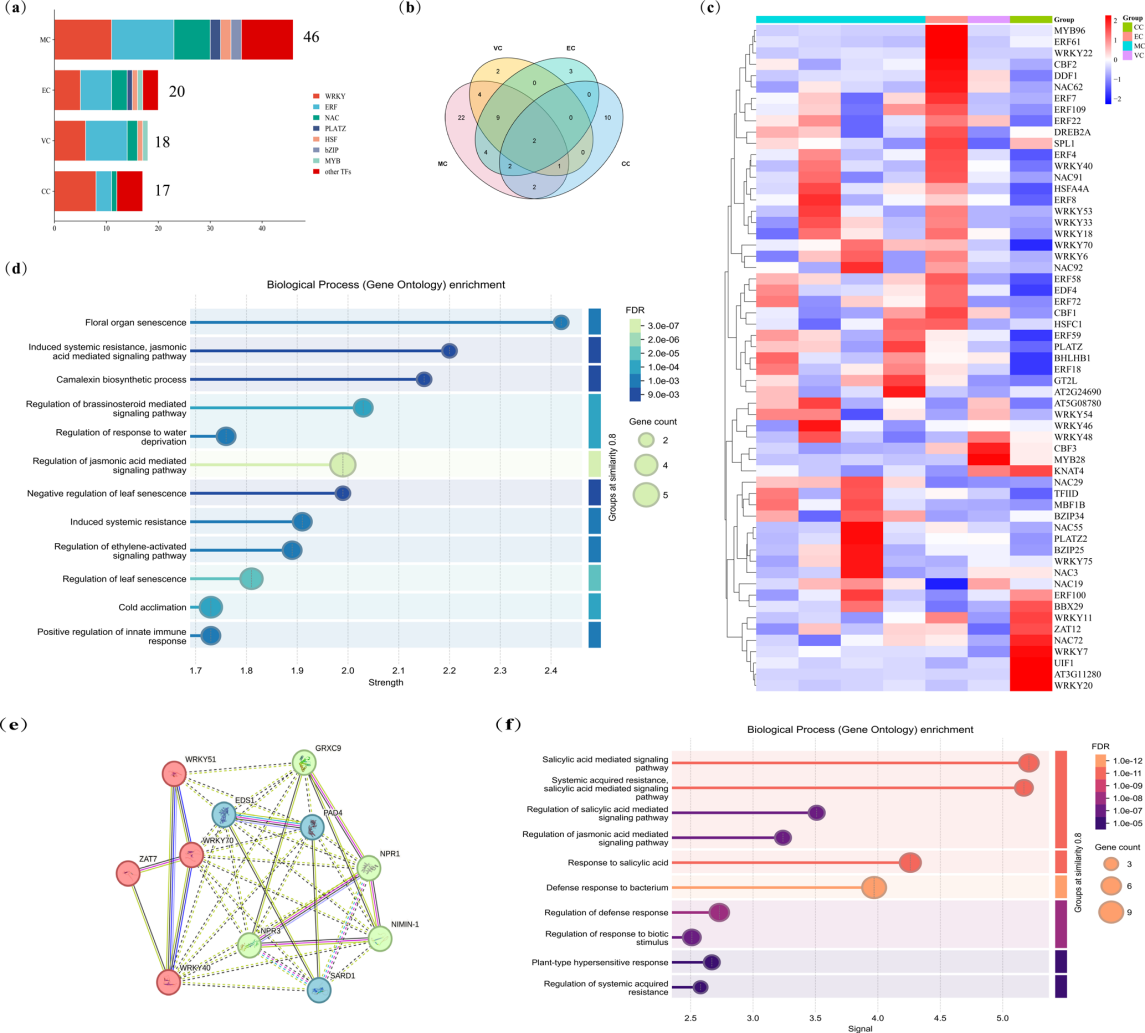
Construction of transcriptional regulatory networks in different cell types. (a) Number of differentially expressed transcription factors (TFs) across all cell types. (b) Venn diagram illustrating the TFs among different cell types. (c) Heatmap of TF expression profiles. (d) GO enrichment analysis of identified TFs. (e) Regulatory network of *AtWRKY70* and its target genes. (f) GO enrichment analysis of target genes regulated by *AtWRKY70*.

Plant stress responses involve complex transcriptional regulatory networks, with transcription factors (TFs) playing pivotal roles in modulating development and environmental adaptation. TFs play a crucial role in responses to biotic stress. To identify cell‐type‐specific and cell‐state‐specific gene regulatory mechanisms, we screened for differentially expressed TFs in our single‐cell data (Table S6). A total of 61 TFs were identified, including members of WRKY, ERF, NAC, BZIP and MYB families, where WRKY and ERF constituted the most abundant families (Figure 5a). In addition, we found that 22 TFs were specifically expressed in MC, 10 in CC, 3 in EC and 2 in VC (Figure 5b,c). Shared TFs across multiple cell types showed GO enrichment for SAR, JA signalling, and innate immune responses (Figure 5d), demonstrating both conserved and divergent regulatory programmes. Intriguingly, *AtWRKY70* and *AtWRKY54* were co‐expressed across all four cell types, suggesting core regulatory functions during PWN infection. *AtWRKY70* and its homologue *AtWRKY54* have been previously identified as key regulators in the induction of immune output genes (Kalde et al. 2003; Wang et al. 2006). STING database analysis predicted *AtWRKY70* target genes predominantly associated with SA signalling (Figure 5e,f), consistent with its role as a primary TF for SA‐mediated transcriptional responses (Powers et al. 2024). These findings further implicate SA‐activated defence pathways in resistance against PWNs.

### 2.5 Functional Analyses of *AtWRKY70* in *Arabidopsis* to Resistance Against PWNs

**FIGURE 6:**
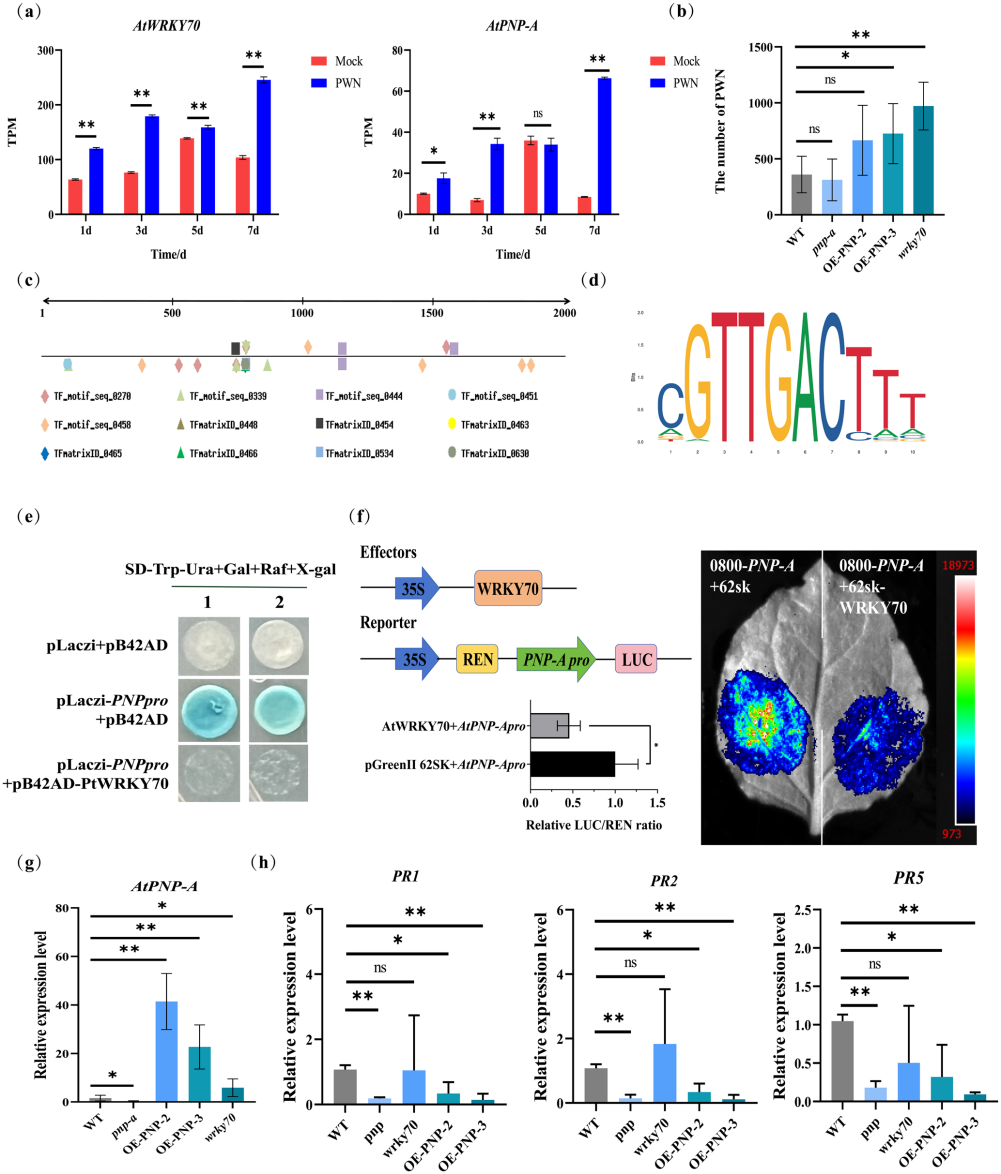
Functional analysis of *AtPNP‐A* and *AtWRKY70* in *Arabidopsis thaliana* resistance against pinewood nematodes (PWNs). (a) TPM of *AtPNP‐A* and *AtWRKY70* at different time points during PWN infection. (b) Statistical analysis of nematode content in PWN inoculation experiments. (c) PlantPAN 3.0 analysis of the *AtPNP‐A* promoter revealed cis‐regulatory elements. (d) AtWRKY70‐binding motif in the *AtPNP‐A* promoter. (e) Yeast one‐hybrid assays demonstrated direct binding of AtWRKY70 to the *AtPNP‐A* promoter region. (f) Dual‐LUC assay showed that AtWRKY70 inhibited the promoter activity of *AtPNP‐A*. (g) Relative expression levels of *AtPNP‐A* in transgenic lines and mutant plants. (h) The expression level of *PR* genes in the mutant during PWN infection. Statistical analysis: ‘ns’ indicates no significant difference, *p < 0.05, **p < 0.01.

To investigate functional roles of AtWRKY70 and SA in *Arabidopsis* resistance against PWNs, we identified plant natriuretic peptide AtPNP‐A—a systemically mobile molecule with a signal peptide domain—from SA‐associated differentially expressed genes. While AtPNP‐A modulates responses to environmental stimuli and its loss enhances SA‐mediated signalling (Meier et al. 2008; Lee et al. 2020), its biological functions remain elusive, particularly during PWN infection. Transcriptomic analysis revealed *AtWRKY70* and *AtPNP‐A* were significantly up‐regulated during PWN infection (Figure 6a). To investigate the roles of *AtPNP‐A* and *AtWRKY70* in PWN infection, we therefore employed *AtPNP‐A* mutants (*pnp‐a*, N681890), overexpression lines (*OE‐PNP‐2/3*) and *AtWRKY70* mutants (*wrky70*, N665593) for PWN inoculation. Although no significant differences in disease incidence were observed among these lines at 3 and 7 days post‐inoculation (Figure S6), the nematode content in *pnp‐a* plants was lower than that in the wild type (WT), whereas *OE‐PNP‐A‐2/3* and *wrky70* lines exhibited significantly higher nematode accumulation (Figure 6b). These results indicated that *AtPNP‐A* could promote PWN infection, while *AtWRKY70* enhances *Arabidopsis* resistance to PWNs.

Intriguingly, *AtPNP‐A* promoter sequence analysis revealed that WRKY binding elements (CGTTGACTTT) were found (Figure 6c,d). Thus, we hypothesised that *AtPNP‐A* could be regulated by AtWRKY70. To test this, yeast one‐hybridisation test and dual‐luciferase reporter assays were performed. These results showed that AtWRKY70 inhibited the activity of the *AtPNP‐A* promoter (Figure 6e,f). Further analyses showed that the expression of *wrky70* was significantly higher than that of WT (Figure 6g), which is consistent with previous studies (Wang et al. 2006; Lee et al. 2020). These results indicated that *AtWRKY70* was up‐regulated to inhibit the expression of *AtPNP‐A* to improve plant resistance to PWN. We also examined the expression of *PR* genes in various mutant plants following PWN infection (Figure 6h). These results indicated that *AtWRKY70* and *AtPNP‐A* regulate PWN infection in an SA‐dependent manner.

## 3 Discussion

Plant immune responses exhibit cellular heterogeneity, requiring precise coordination of different cell types at specific times and locations. The application of single‐cell transcriptomics has enabled the resolution of plant immune processes at the individual cell level (Liu et al. 2021; Chen et al. 2025). Previous cell atlases have made significant progress in cell type identification (Kim et al. 2021; Xia et al. 2022). However, the use of scRNA‐seq to understand PWN infections of plant hosts has been rare to date.

In this study, we used snRNA‐seq to identify genes crucial for PWN infection. We discovered that distinct cell types contribute to immunity through different pathways: vascular cells primarily engage with the JA signalling pathway, epidermal cells are significantly enriched in the SA signalling pathway, and mesophyll cells are associated with reactive ROS and secondary metabolites (Figure 2d,e). While the regulatory mechanisms of SA and JA signalling pathways in plant immunity have been extensively reported (Roychowdhury et al. 2024; Vlot et al. 2009; Mishra et al. 2020), the critical biological roles of secondary metabolites in mediating plant responses to biotic and abiotic stressors have gained increasing attention in recent years (Anjali Kumar et al. 2023; Yang et al. 2012; Aljbory and Chen 2018). While current insights into plant–pathogen interactions predominantly derive from bulk transcriptome analyses of whole tissues, single‐cell transcriptomics has uncovered the compartmentalised roles of epidermal cells, vascular bundles and mesophyll cells in pathogen defence. Our findings resonate with cell‐type‐stratified immune dynamics observed across plant species. For instance, Liang et al. (2023) reported divergent GO enrichment profiles among *Hevea brasiliensis* cell types during powdery mildew infection, with immune pathways markedly down‐regulated in epidermal and meristematic cells, and proposed that the host's response to the pathogen is a variable process. Similarly, Zha et al. (2023) demonstrated that brown planthopper resistance in rice arises not from a single phloem‐localised factor but from synergistic interactions of resistance determinants spatially distributed across multiple cell types. This collective evidence underscores the necessity to dissect crosstalk between compartmentalised immune pathways, which will advance mechanistic understanding of the spatiotemporal regulatory networks governing host–pathogen coevolution.

To further elucidate cell‐type‐specific responses, our subcluster analyses revealed that *Arabidopsis* response to PWN infection is predominantly localised in mesophyll and epidermal cell clusters (Figure 3d; Figure S3). In this study, immune responses were observed in all cell types; this may be attributed to the long‐distance transport of immune signals. Meanwhile, our analysis reveals a spatially compartmentalised immune response architecture in plant cells, manifested through distinct cellular states. Specifically, we identified immune‐active subpopulations primarily localised to clusters 1, 3 and 5 in epidermal cells (Figure 3d,e), while mesophyll cell immunity was predominantly associated with clusters 2 and 5. Pseudotime trajectory reconstruction (Figure 4b–e) delineating the continuum from non‐immune → transitional → immune cell populations further demonstrated the asynchronous nature of plant responses to pathogen invasion. The host immune response exhibits dynamic shifts between active defence mechanisms and passive adaptation strategies as pathogen infection progresses. This phased response mirrors the pathogen's ability to induce differential host pathways depending on infection intensity, consistent with previous reports of asynchronous infection stages in plant–pathogen interactions (Fantozzi et al. 2021). Notably, single‐cell profiling of *Pseudomonas syringae* ‐infected *Arabidopsis* leaves similarly revealed coexisting cell clusters exhibiting antagonistic biological processes (immunity vs. susceptibility) at 24 h post‐inoculation, further validating the concept of cell state heterogeneity during early infection (Zhu et al. 2023). Analysis of genes highly expressed in immune cell populations may help us identify new immune marker genes. For example, Nobori et al. (2025) identified a novel cellular state and also identified the PRIMER cell marker gene *GT‐3A*, which has also been shown to contribute to the immunity of plants against pathogen infections.

TFs play a pivotal role in regulating plant responses to both biotic and abiotic stresses (Haddoudi et al. 2025; Wani et al. 2021; Yue et al. 2024). In this study, we identified key TFs, including WRKY and ERF families, involved in *A. thaliana* 's response to PWN infection. These TFs can bind to the promoters of PR protein genes, thereby modulating plant defence responses (Luo et al. 2005; Pei et al. 2024; Yu et al. 2018). We found that *AtWRKY70* plays a central regulatory role in plant resistance to PWNs (Figure 6). *AtWRKY70* exhibits complex roles in plant immunity, including both negative effects on SA biosynthesis and positive roles in SA signalling (Li et al. 2004, 2006; Hu et al. 2012; Wang et al. 2006). Notably, our study revealed that AtWRKY70 binds to the *AtPNP‐A* promoter and represses its expression (Figure 6d,e). This finding is particularly significant as previous reports demonstrated that *XacPNP* counteracts SA‐mediated immune responses to facilitate bacterial colonisation (Gottig et al. 2008). In addition, *AtPNP‐A* has been found to negatively regulate plant resistance (Lee et al. 2020). We also found that *AtPNP‐A* functions as a negative regulator of *Arabidopsis* resistance to PWNs (Figure 6c). *AtPNP‐A* antagonises SA‐triggered immunity to prevent excessive stress responses that could lead to cellular necrosis (Lee et al. 2020). Thus, AtWRKY70 enhances SA‐mediated immunity mainly through suppressing the negative regulatory function of *AtPNP‐A*. However, further experimental evidence is required to elucidate how AtWRKY70‐mediated transcriptional regulation of *AtPNP‐A* participates in SA‐mediated plant immune responses and whether AtWRKY70 influences *AtPNP‐A* function at the post‐transcriptional level.

Investigating the defence mechanisms of host pine trees against PWN infection is crucial for understanding the intrinsic causes of pine tree mortality and identifying effective control strategies (Wang et al. 2023; Modesto et al. 2022; Liu, Zhang, Fang, et al. 2022; Liu, Zhang, Xin, et al. 2022). However, research on pine trees as host plants has been limited, and the molecular‐level understanding of the interactions between pine trees and PWNs remains incomplete. Given the crucial roles of these genes in *Arabidopsis*–PWN interactions, we identified their putative orthologues in *Pinus tabuliformis* —*PtWRKY60* (*Pt5G34100, AtWRKY70* orthologue) and *PtPNP‐A* (*Pt5G28510, AtPNP‐A* orthologue). Remarkably, these pine genes exhibited conserved expression patterns during PWN infection (Figure S7). This evolutionary conservation strongly suggests that the *WRKY70–PNP‐A* regulatory module plays a critical role in plant resistance against PWNs. It also provides a possibility for *A. thaliana* to be used as a research model to study the interaction mechanism between PWNs and their host.

In summary, we constructed a cellular atlas of the immune response in PWN‐infected *A. thaliana* leaves. Our results demonstrated that PWN‐induced transcriptional reprogramming varies significantly across different cell types. We delineated the immune states of cell populations and identified the critical role of the AtWRKY70–AtPNP‐A transcriptional regulatory module during PWN infection. These findings provide valuable insights into the intricate interactions between the PWN and its host, offering a foundation for further understanding the molecular mechanisms underlying plant–pathogen dynamics.

## 4 Experimental Procedures

### 4.1 Gene Symbols, Names and Ordered Locus Names

The following genes were highlighted in this study: system‐acquired resistance immune genes: *PR1* (*AT2G14610*), *PR2* (*AT3G57260*), *PR5* (*AT1G75040*), *SAG13* (*AT2G29350*), *CBP60g* (*At5g26920*), *PDF1.2c* (*AT5G44430*); pattern recognition receptors (PRR)‐triggered immunity (PTI) pathway genes: *CRK4* (*AT3G45860*), *CRK7* (*AT4G23150*), *FOX1* (*AT1G26380*), *RMG1* (*AT4G11170*), *FMO1* (*At1g19250*) (Zhu et al. 2023; Idänheimo et al. 2014; Lang et al. 2022; Sharma and Prasad 2024; Sistenich et al. 2024); JA signal transduction pathway genes: *CHIL* (*AT5G05270*), *CLH1* (*AT5G05270*), *OPR3* (*AT2G06050*), *ML3* (*AT5G23820*); Insect resistance gene: *CYP79B2* (*AT4G39950*), *DEFL206* (*AT3G59930*), *LTPG5* (*AT3G22600*), *LipoP1* (*AT3G18250*), *VSP2* (*AT5G24770*).

### 4.2 Plant Growth and Inoculation

*Nicotiana benthamiana* and *Arabidopsis* plants were grown in a growth chamber (24°C:22°C, day:night, 16 h:8 h, light:dark) for subsequent inoculation and infiltration experiments. The PWN (*Bursaphelenchus xylophilus*) used in this study was isolated from naturally infected Korean pine (*Pinus koraiensis*) branches in Shenyang, Liaoning Province, China. Nematodes were extracted using the Baermann funnel method (Feng 2005) and cultured on *Botrytis cinerea*‐potato dextrose agar (PDA) medium at 25°C for 10 days. The nematodes were then washed three times with sterile distilled water, collected in 1.5 mL microfuge tubes, and resuspended in sterile water. A 10 μL aliquot of the nematode suspension was placed on a clean glass slide and counted under a low‐power microscope (Wang et al. 2023). This process was repeated 10 times, and the average count was used to estimate the total number of nematodes collected.

The inoculation method was adapted from Jian et al. (2012) with modifications. Briefly, a nematode suspension was adjusted to a concentration of 600 nematodes/10 μL. *Arabidopsis* plants, grown for 22 days, at which point *A. thaliana* contained 8–9 rosette leaves, were gently wounded near the base of the petiole using sterile forceps. A 10 μL aliquot of the nematode suspension was then applied to the wound site. After inoculation, the trays were covered with a transparent lid, and sterile water was added every 2–3 h to maintain moisture at the wound site for 12 h. The lid was removed after 24 h. Control plants were inoculated with sterile water.

### 4.3 Nuclei Isolation and snRNA‐Seq

Leaves from *Arabidopsis* plants inoculated with PWNs for 7 days (BX) and control leaves (CK) were collected for single‐nucleus RNA sequencing (snRNA‐seq). Isolation of nuclei was performed as previously described (Thibivilliers et al. 2020; Liu, Su, et al. 2023; Liu, Yang, et al. 2023). The nuclear suspension was prepared using the DG1000 system (BioMarker Technologies) for reverse transcription of mRNA and cDNA library construction. Two biological replicates were performed for each sample.

### 4.4 Single‐Nuclei Data Analysis

Raw snRNA‐seq data were processed using Cell Ranger 3.0.2 (10× Genomics) for quality control and mapping to the *Arabidopsis* genome (TAIR 10). Further quality control and normalisation were performed using the Seurat package (v. 3.0) for downstream analysis (Zhu et al. 2023). Marker genes for each cell cluster were identified using the Find All Markers function, with screening criteria set at an adjusted *p*‐value less than 0.01 and a log_2_FC greater than 0.5 (corresponding to FC > 1.414). The pseudo‐bulk gene expression matrices of cell subtypes were extracted using the Seurat package. Pearson correlation coefficients between different cell subtypes were calculated using the cor function. Subsequently, heatmaps or scatter plots of the correlation coefficients were generated using the ggplot2 package.

### 4.5 DEGs and Functional Enrichment Analysis

DEGs were identified using the Seurat package, with a significance threshold of *p* ≤ 0.01 and log_2_FC ≥ |0.5| (Tables S3 and S4). Gene Ontology (GO) enrichment was performed using the R package clusterProfiler.

### 4.6 Immune Response Score for PWNs

The immune response score (IRS), defined as a composite metric quantifying a cell's general state in disease progression based on gene sets associated with induced immunity or late‐stage susceptibility (Zhu et al. 2023), was calculated. To identify PWN‐responsive immunity‐associated and susceptibility‐associated genes, we curated published plant immunity multi‐omics datasets and established immune marker genes, integrating these with DEGs identified between PWN‐inoculated and Mock samples. Following Zhu et al.'s methodology for selected genes (Table S5), immunity and susceptibility module scores were computed using the AddModuleScore function (Seurat). To further identify and validate PWN‐responsive immunity genes, time‐course bulk RNA sequencing (RNA‐seq) was performed on leaves harvested at 1, 3, 5 and 7 dpi with PWN.

### 4.7 Pseudotime Trajectory Analysis and Regulatory Network

Pseudotime trajectory analysis of single‐cell transcriptomes was conducted using Monocle 3. Cluster‐specific genes were identified using the differential GeneTest function (Qiu et al. 2017). These genes were used to reconstruct cell trajectories in Seurat. Heatmaps of the average expression of highly variable genes within each cluster were generated, and the data were subsequently used for GO biological process analysis. TFs and their target genes are available from the STING database (https://cn.string‐db.org) and were visualised using Cytoscape network maps. Prediction of promoter binding sites was performed using JASPA (http://jaspardev.genereg.net/). The homologous genes of *Pinus tabulaeformis* were obtained by the sequence of AtWRKY70 and AtPNP‐A proteins and coding sequences (CDS) in the genome BLAST (http://conifers.cn/CperProject/front/#/blast).

### 4.8 Dual‐Luciferase Reporter Assay

The full‐length CDS of *AtWRKY70* was cloned into the pGreenII 62‐SK vector, while the 2000 bp promoter regions of *AtPNP‐2A* were cloned into the pGreenII 0800‐LUC vector. The cloning primers are shown in Table S7. The pGreenII 62‐SK empty vector was used as a negative control. *Agrobacterium tumefaciens* GV3101 (pSoup) carrying the constructs was infiltrated into *N. benthamiana* leaves. After 3 days, leaves were sprayed with 1 mM luciferin substrate and incubated in the dark for 10–15 min before imaging using a precooled CCD camera. The samples were ground in liquid nitrogen, mixed with 100 μL of reporter lysis buffer, and centrifuged at 10,000–15,000 *g* for 3–5 min. The supernatant was collected, and firefly luciferase activity was measured using the Dual‐Luciferase Reporter Assay Kit (RG 027; Beyotime Biotechnology). Renilla luciferase activity was used as an internal control, and the LUC/REN ratio was calculated. Three biological replicates were performed for each sample.

### 4.9 Yeast One‐Hybrid Assays

The promoter sequence of AtPNP‐A was cloned into the pLacZ2u vector, and the full‐length CDS of *AtWRKY70* was cloned into the pB42AD vector (primers listed in Table S7). Both constructs were co‐transformed into yeast strain EGY48, using empty vectors as negative controls. Transformants were selected on SD/−Trp/−Ura medium and incubated at 28°C for 3–5 days. Individual colonies were then spotted onto SD/−Trp/−Ura medium supplemented with galactose/raffinose (Gal/Raf) and X‐gal chromogenic substrate. Following incubation at 28°C for 3–5 days, β‐galactosidase activity was assessed.

### 4.10 Transformation of Transgenic Lines

The CDS of *AtWRKY70, AtPNP‐2A* (without stop codons) were cloned into the PGAM1300 vector with a FLAG tag and transformed into *A. tumefaciens* GV3101. The cloning primers are shown in Table S7. Transgenic *Arabidopsis* lines were generated using the floral dip method (Xu et al. 2021). Briefly, 0.1 mL of *Agrobacterium* culture was inoculated into 50 mL of liquid Luria Bertani (LB) medium containing the appropriate antibiotics and grown at 28°C for 12 h. The cells were pelleted by centrifugation at 4000 rpm for 10 min and resuspended in infiltration buffer (5% sucrose, 20% vol/vol Silwet L‐77) to an OD_600_ of 0.8–1.0. *Arabidopsis* inflorescences were dipped into the suspension for approximately 45 s, and young flower buds were treated with the suspension using a pipette. The plants were kept in the dark for 24 h before being transferred to normal growth conditions (22°C–25°C, light). T_0_ seeds were harvested, dried for 3–5 days, and screened on solid Murashige and Skoog (MS) medium containing the appropriate antibiotic (34 mg/L hygromycin). The mutant was identified as a homozygous strain by the ‘three‐primer identification method’, and the primer sequences are shown in Table S7.

### 4.11 RT‐qPCR Analysis

To determine transcript levels in mutant and overexpression lines, leaves were harvested from respective transgenic plants. Three independent biological replicates were processed for RT‐qPCR analysis. To validate the applicability and expression dynamics of selected immune‐responsive marker genes in the *A. thaliana–B. xylophilus* pathosystem, leaves were collected at 1, 3, 5 and 7 dpi with nematodes. Mock‐inoculated controls (distilled water treatment) were processed in parallel. Three biological replicates were set for each sample. Transcript levels were quantified by RT‐qPCR. Primer sequences are provided in Table S7.

### 4.12 Statistical Analysis

All statistical analyses were performed using SPSS software. Student's *t* test was used to calculate *p* values, with statistical significance determined at ‘ns’ indicates no significant difference; *p < 0.05, **p < 0.01 levels. Statistical data plots were generated using GraphPad.

## Author Contributions

**Meiling Wang:** conceptualisation; investigation; methodology; formal analysis; data curation; writing – original draft; visualisation; writing – review and editing. **Xiehai Song:** conceptualisation; formal analysis; data curation; writing – review and editing; methodology; visualisation. **Zhiyuan Jiao:** conceptualisation; supervision; writing – original draft; writing – review and editing. **Jiashu Zhang:** methodology. **Yue Sang:** methodology. **Wei Li:** conceptualisation; supervision; funding acquisition; resources; writing – original draft; methodology; project administration.

## Conflicts of Interest

The authors declare no conflicts of interest.

## Supporting information


**Figure S1:** UMAP maps of two biological replicates of Mock and PWN samples. (a) Mock_rep 1; (b) Mock_rep 2; (c) PWN_rep 1; (d) PWN _rep 2.

**Figure S2:** Subtype classification and GO enrichment analysis of vascular cells and companion cells. (a) Vascular cell subtypes of Mock; (b) vascular cell subtypes of PWN; (c) GO enrichment analysis of vascular cell subtypes; (d) companion cell subtypes of Mock; (e) companion cell subtypes of PWN; (f) GO enrichment analysis of companion cell subtypes.

**Figure S3:** The expression level of epidermal immune marker genes. (a) Transcripts per million (TPM) of epidermal immune marker genes. (b) The relative expression levels of epidermal immune marker genes were verified by RT‐qPCR.

**Figure S4:** Specific response of mesophyll cell subclass to PWN. (a) UMAP of mesophyll cell subtypes; (b) the proportion of clusters of mesophyll cell subtypes; (c) the ratio of each cell subtypes of PWN and Mock; (d) heat map of the expression of the top 10 genes specifically expressed for each cluster in the mesophyll; (e) GO enrichment analysis of genes within each cluster.

**Figure S5:** The expression level mesophyll immune marker genes. (a) Transcripts per million (TPM) of mesophyll immune marker genes; (b) the relative expression levels of mesophyll immune marker genes were verified by RT‐qPCR.

**Figure S6:** Phenotype and incidence of PWN‐inoculated in Arabidopsis leaves. Phenotype (a) and incidence (b) of PWN‐inoculated in *Arabidopsis* leaves.

**Figure S7:** TPM of *WRKY70* and *PNP‐A* homologous genes of *Pinus tabulatus* after inoculation with PWN. (a) The expression of *WRKY70* homologous gene of *Pinus tabulaeformis*; (b) the expression of *PNP‐A* homologous gene of *P. tabulaeformis*.

**Table S1:** Statistical table of sequencing.

**Table S2:** List of cell‐type marker genes used in this study.

**Table S3:** List of DEGs of different cell types.

**Table S4:** List of marker genes of epidermal and mesophyll cell subtypes.

**Table S5:** The list of genes used in the immune response scoring.

**Table S6:** List of differentially expressed transcription factors.

**Table S7:** List of primers used in the article.

## Data Availability

The data that supports the findings of this study are available in the Supporting Information of this article. The sequence data have been deposited in the China National Center for Bioinformation (PRJCA038339).
