# Supplementary figures and images for: snRNA‐Seq Unveils Cell‐Type‐Specific Immune Dynamics in Arabidopsis During Pinewood Nematode Infection
Source: Mol Plant Pathol. 2025 Aug 18;26(8):e70136. doi: 10.1111/mpp.70136 (PMC12361641; doi:10.1111/mpp.70136)

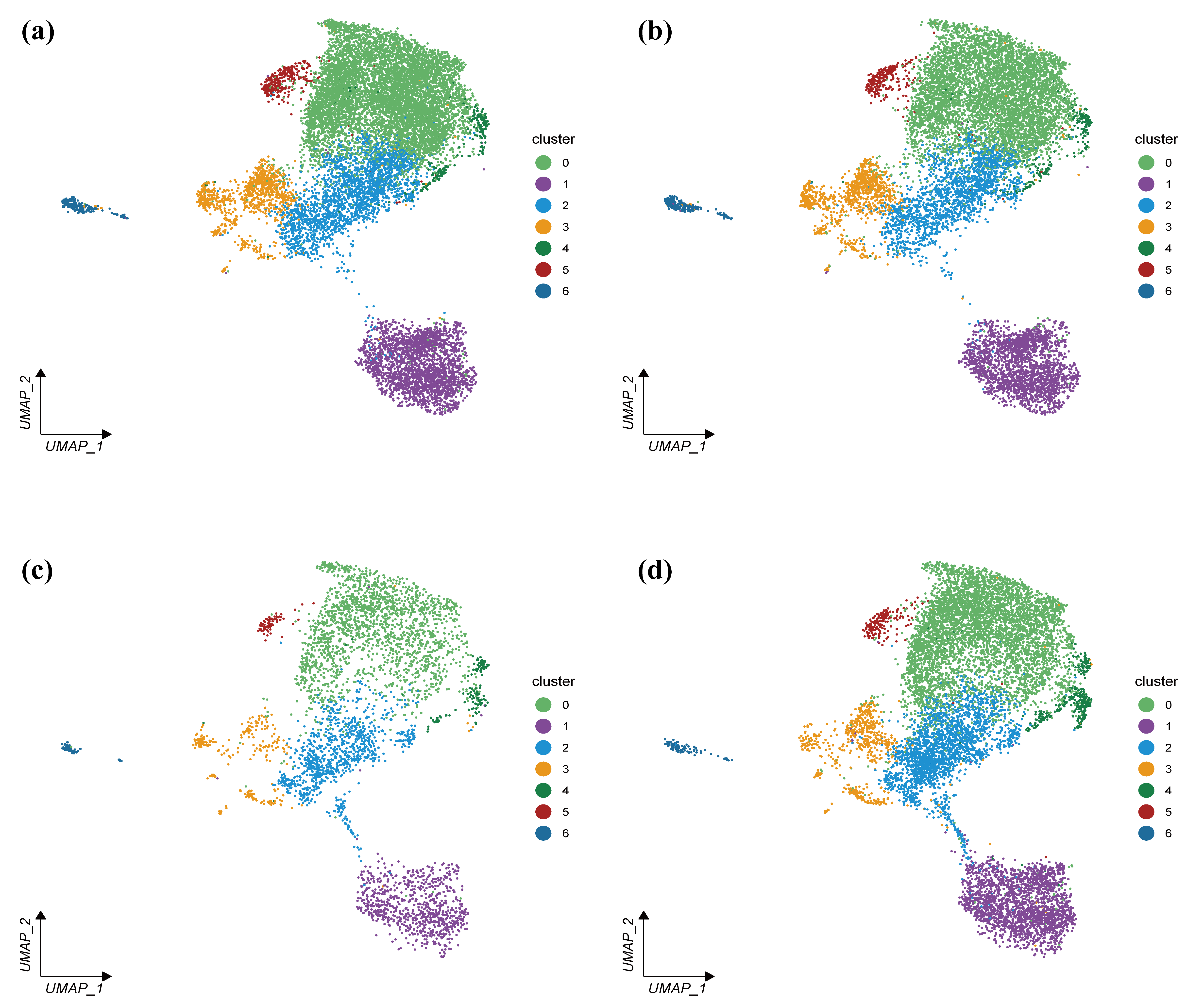

Supplement: Supplementary file 1 — Figure S1: UMAP maps of two biological replicates of Mock and PWN samples. (a) Mock_rep 1; (b) Mock_rep 2; (c) PWN_rep 1; (d) PWN _rep 2. [file MPP-26-e70136-s008.png]

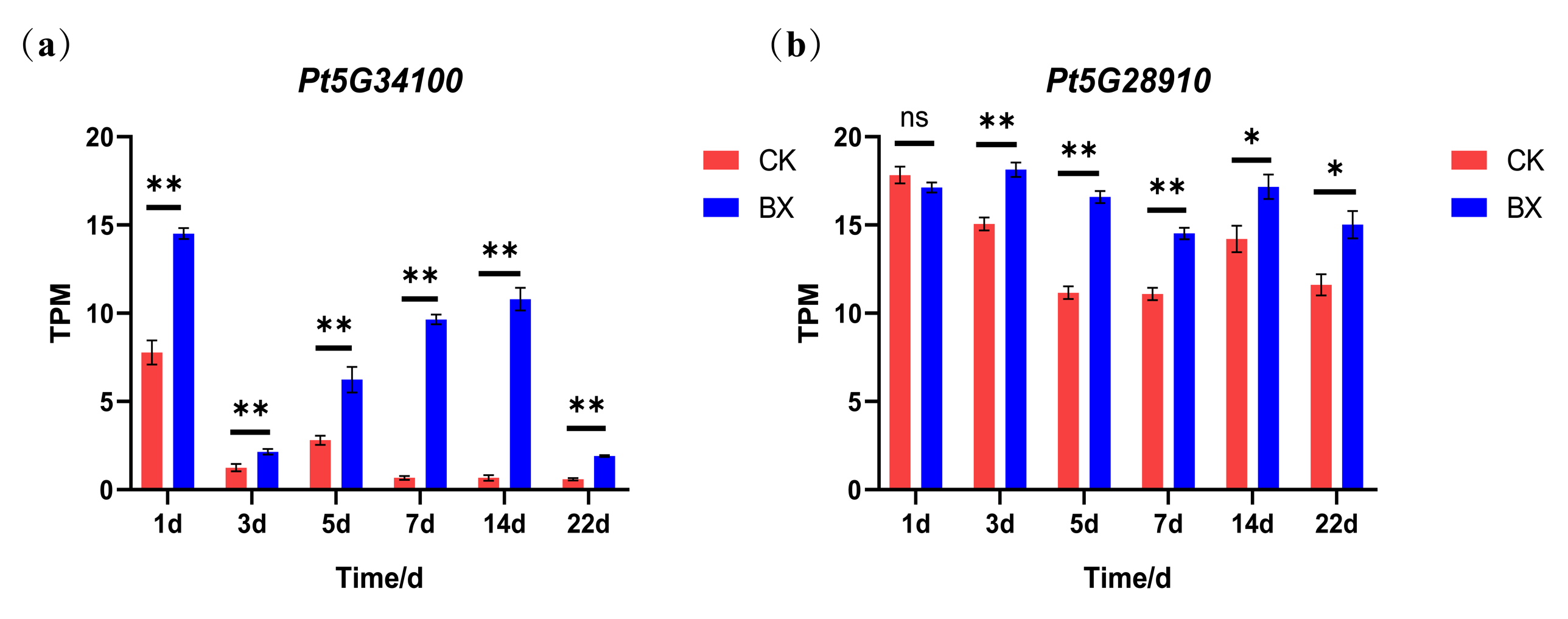

Supplement: Supplementary file 7 — Figure S7: TPM of WRKY70 and PNP‐A homologous genes of Pinus tabulatus after inoculation with PWN. (a) The expression of WRKY70 homologous gene of Pinus tabulaeformis; (b) the expression of PNP‐A homologous gene of P. tabulaeformis. [file MPP-26-e70136-s012.png]
